# DNA methylation of the *RUNX2* P1 promoter mediates *MMP13* transcription in chondrocytes

**DOI:** 10.1038/s41598-017-08418-8

**Published:** 2017-08-10

**Authors:** Atsushi Takahashi, María C. de Andrés, Ko Hashimoto, Eiji Itoi, Miguel Otero, Mary B. Goldring, Richard O. C. Oreffo

**Affiliations:** 1 Bone and Joint Research Group, Centre for Human Development Stem Cells and Regeneration, Institute of Developmental Science, University of Southampton Medical School, Southampton, UK; 2 Department of Orthopaedic Surgery, Tohoku University School of Medicine, Sendai, Japan; 3 HSS Research Institute, Hospital for Special Surgery, and Weill Cornell Medical College, New York, NY USA

## Abstract

The Runt-related transcription factor 2 (RUNX2) is critical for bone formation as well as chondrocyte maturation. Matrix metalloproteinase (MMP)-13 is a major contributor to cartilage degradation in osteoarthritis (OA). We and others have shown that the abnormal *MMP13* gene expression in OA chondrocytes is controlled by changes in the DNA methylation status of specific CpG sites of the proximal promoter, as well as by the actions of different transactivators, including RUNX2. The present study aimed to determine the influence of the methylation status of specific CpG sites in the *RUNX2* promoter on RUNX2-driven *MMP13* gene expression in OA chondrocytes. We observed a significant correlation between *MMP13* mRNA levels and *RUNX2* gene expression in human OA chondrocytes. RUNX2 overexpression enhanced *MMP13* promoter activity, independent of the *MMP13* promoter methylation status. A significant negative correlation was observed between *RUNX2* mRNA levels in OA chondrocytes and the percentage methylation of the CpG sites in the *RUNX2* P1 promoter. Accordingly, the activity of the wild type *RUNX2* promoter was decreased upon methylation treatment *in vitro*. We conclude that *RUNX2* gene transcription is regulated by the methylation status of specific CpG sites in the promoter and may determine *RUNX2* availability in OA cartilage for transactivation of genes such as *MMP13*.

## Introduction

The Runt-related transcription factor 2 (RUNX2) is critical for osteoblast differentiation and bone formation^1–3^. Mice with a homozygous mutation in *Runx2* display a complete lack of ossification in their skeletal systems^4^, while *RUNX2* haploinsufficiency presents as the autosomal dominant skeletal disorder, cleidocranial dysplasia^5, 6^. RUNX2 is also essential for chondrocyte maturation^7–11^. Matrix metalloproteinase *(MMP)-13* gene expression is decreased in *Runx2*-null mutant mice^12–14^, and Runx2 interacts with Osterix in regulating *MMP13* gene transcription in growth plate chondrocytes^15^. Importantly, both *Runx2*-deficient mice and chondrocyte-specific *Runx2*–transgenic mice display abnormal cartilage development^8, 9^, and *Runx2*-haploinsufficient mice show reduced type X collagen and MMP13 protein and mRNA levels accompanied by decreased cartilage degradation in osteoarthritis (OA) models^16^. Furthermore, the dominant-negative form of RUNX2 severely inhibits alkaline phosphatase activity and matrix calcification in mature chondrocytes, while retroviral transduction of RUNX2 in chick immature chondrocytes induces type X collagen gene expression, alkaline phosphatase activity, and extensive cartilage-matrix mineralization^7, 11^.

RUNX2 exists as two isoforms initiated from two different promoters, the distal P1 promoter and the proximal P2 promoter. P1 and P2 are separated by exon 1 and a large intron^3^. Type II isoform transcription is initiated at the distal promoter P1, whilst type I isoform transcription starts at the proximal promoter P2^17^. Interestingly, the two proteins differ in only 19 amino acids at the N terminus, share the same functional domains, and are capable of trans-activating target genes^18, 19^. Type I RUNX2 was originally cloned as a T-cell specific factor (Pebp2αA), and type II as a bone-specific factor (Osf-2)^20–23^. The type I isoform is expressed in non-osseous mesenchymal cells, osteoprogenitors, chondrocytes, and thymocytes, while the type II isoform is restricted to osseous cells and mature chondrocytes of the developing axial skeleton^1, 18^.

DNA methylation at CpG sites is a major epigenetic mechanism by which cells maintain dominant phenotypes and stable chromatin configurations, conferring long-term regulation of specific genes in contrast to histone modifications, which are more dynamic and reversible^24–26^. Altered DNA methylation patterns are associated with abnormal gene expression in several pathologies, including OA^27–29^, and although DNA methylation of the CpG islands has been studied extensively, recent evidence indicates that the methylation status of specific CpG sites can also alter gene expression by impacting promoter or enhancer activities and the DNA binding of specific transcription factors^28–31^. Importantly, a 0.6 kb sequence upstream of the transcription start site (TSS) is sufficient for driving RUNX2 P1 promoter activity^3, 32^. While only a few CpG sites are present within this upstream region of the P1 promoter, little is known about the specific impact of the methylation status of these CpG sites on gene control.

OA is a complex, multifactorial disorder triggered by biomechanical and biochemical factors and involving maladaptive repair responses^33–35^. MMP-13 is a major contributor to cartilage degradation in OA disease^36^, by targeting types II and IX collagen^37^. Previous studies have shown enhanced gene expression of *MMP13* in OA cartilage^28, 36, 38^, and that while post-natal overexpression of active MMP13 *in vivo* leads to OA-like changes^39^, knockdown^40^ or knockout^36, 41^ of MMP13 delays the development of experimentally-induced OA. We and others have shown that the abnormal *MMP13* gene expression in OA chondrocytes is controlled by changes in the DNA methylation status of specific CpG sites of the proximal promoter^28, 42^, as well as by the actions of different transactivators^28, 43, 44^, including RUNX2^36, 45, 46^.

We hypothesized that the altered methylation status of specific CpG sites in the P1 promoter of *RUNX2* determines the availability of the expressed gene product, which in turn influences the levels of *MMP13* gene expression in human OA chondrocytes.

## Results

### High *MMP13* gene expression in OA chondrocytes is associated with demethylation of specific CpG sites in the *MMP13* proximal promoter

**Figure 1:**
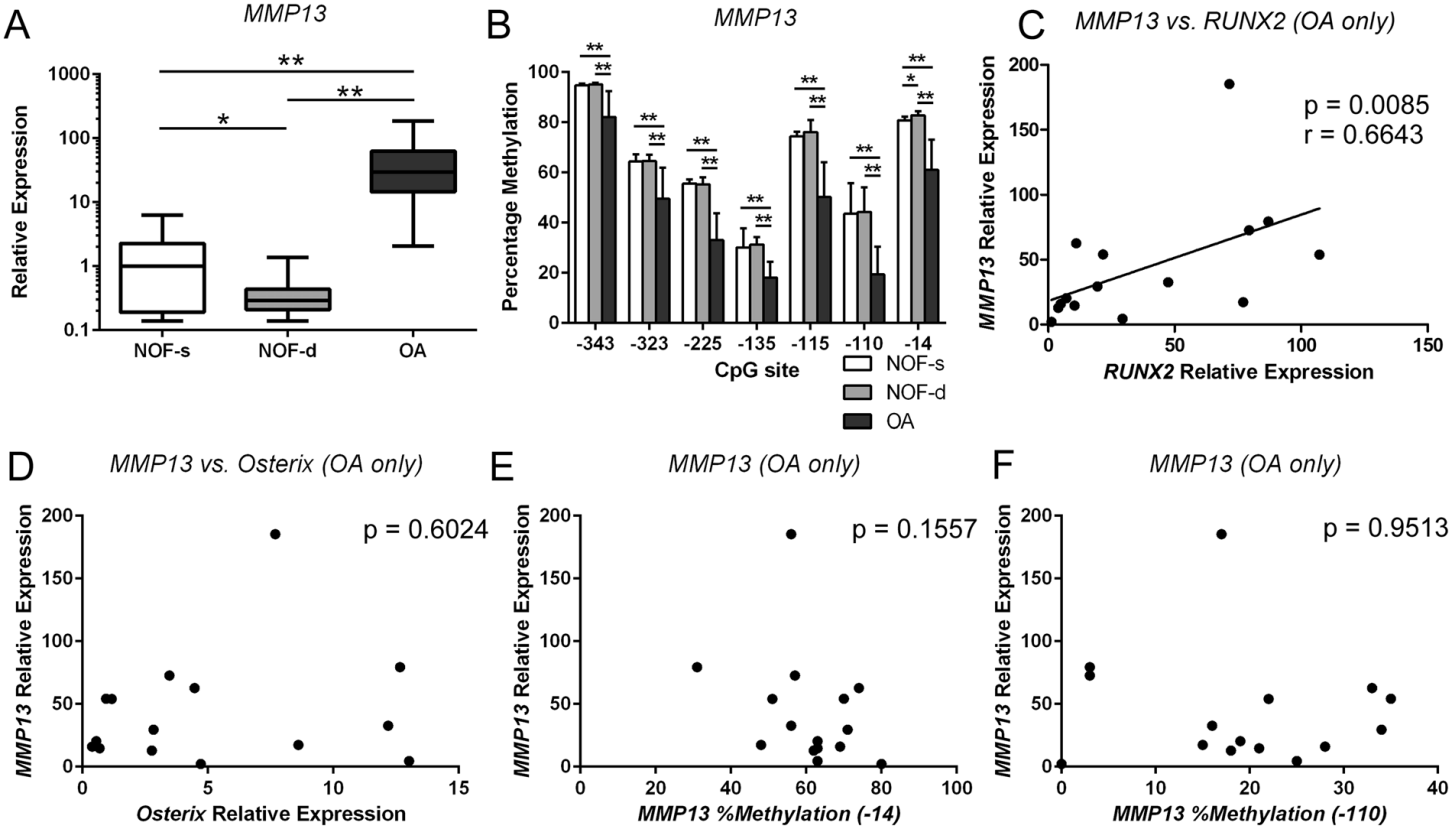
*MMP13* is highly expressed in OA chondrocytes with accompanying demethylation in the CpG sites of the *MMP13* proximal promoter and is associated with *RUNX2* but not *OSX* gene expression or *MMP13* DNA methylation status. Non-cultured primary human chondrocytes were isolated from cartilage obtained from patients with femoral neck fracture (NOF) and OA patients. (**A**) Relative *MMP13* mRNA levels were analysed separately in chondrocytes from the superficial (NOF-s) and deep zones (NOF-d) of NOF cartilage by qRT-PCR and normalized against GAPDH. (**B**) Percentage methylation of each indicated CpG site in the *MMP13* proximal promoter was analysed in the same samples by bisulfite pyrosequencing. The y-axis shows non-adjusted percentage methylation. (**C**–**F**) *MMP13* gene expression in OA chondrocytes was compared with the levels of *RUNX2* mRNA (**C**) or *OSX* mRNA (**D**), and with the methylation status of the −14 bp (**E**) or −110 bp CpG site in the MMP13 proximal promoter (**E**,**F**). Values are the mean ± SD. *P < 0.05, **P < 0.01.

Human articular cartilage was dissected from femoral heads obtained from patients undergoing hemiarthroplasty for fractured neck of femur (NOF, non-OA controls) or from OA patients undergoing total hip arthroplasty. In agreement with previous reports^28, 38^ the level of *MMP13* mRNA in NOF chondrocytes (n = 11 donors) was 3.9-fold higher in the superficial zone than in the deep zone (p < 0.05). In OA chondrocytes (n = 15 donors) *MMP13* mRNA levels were 107-fold higher than in NOF deep chondrocytes (p < 0.01) (Fig. 1A). Pyrosequencing analysis of the proximal *MMP13* promoter in the same subjects revealed that the CpG sites in the analyzed promoter region were significantly demethylated in OA chondrocytes compared to NOF chondrocytes (Fig. 1B), in agreement with previous reports^28, 38^. Only the −14 bp CpG site showed significant differences in the methylation status between superficial and deep zone NOF chondrocytes.

### Enhanced *MMP13* mRNA levels in OA chondrocytes are correlated with *RUNX2*, but not with *OSX*, gene expression or with the DNA methylation status of individual CpG sites on the proximal *MMP13* promoter

To address the relative contribution of selected transactivators to the increased *MMP13* gene expression in OA chondrocytes^15, 28, 46, 47^, we analyzed the correlation between *MMP13* expression levels with the levels of *RUNX2* and *OSX* mRNA and with the CpG methylation status of specific CpG sites in the proximal *MMP13* promoter. As shown in Fig. 1, we observed a significant correlation between *MMP13* and *RUNX2* mRNA levels in OA chondrocytes (p < 0.01) (Fig. 1C), but not in NOF chondrocytes (data not shown). Although it has been shown that *MMP13* is an important target of Osterix^15, 28^, we did not observe a correlation between *MMP13* and *OSX* mRNA levels (Fig. 1D). In addition, although an association between differential methylation of −110 CpG site and *MMP13* promoter activity has been observed by us and others^28, 31^, we did not find a significant correlation between *MMP13* mRNA levels and the methylation status of the −110-bp CpG site or the −14-bp CpG site in this study (Fig. 1E).

### Increased DNA demethylation at CpG sites in the *RUNX2* P1 promoter of OA chondrocytes compared to NOF chondrocytes

**Figure 2:**
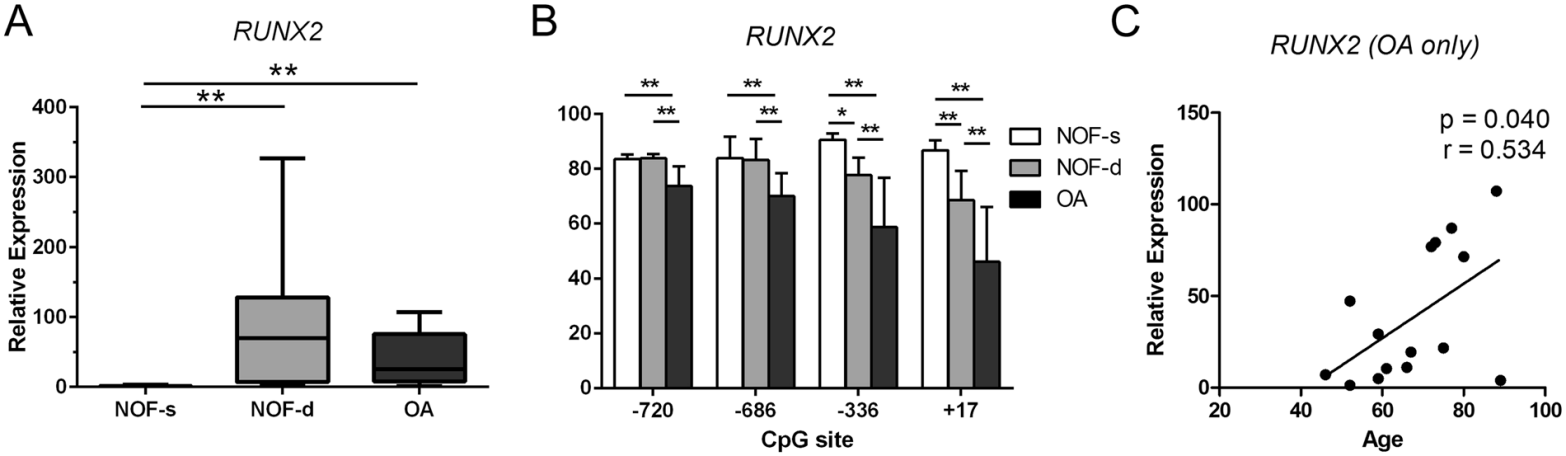
Higher levels of *RUNX2* mRNA in deep zone NOF and OA chondrocytes than in superficial zone NOF chondrocytes are associated with the methylation status of specific CpG sites in the *RUNX2* P1 promoter. (**A**) Relative mRNA levels of *RUNX2* in the superficial (NOF-s) and deep zone (NOF-d) of NOF cartilage and OA cartilage were analysed by qRT-PCR and normalized against GAPDH. (**B**) Percentage methylation of the indicated CpG sites in the *RUNX2 P1* promoter were analysed in genomic DNA simultaneously extracted from the same subjects by bisulfite pyrosequencing. The y-axis indicates non-adjusted percentage methylation. (**C**) *RUNX2* mRNA levels in OA chondrocytes are plotted against the ages of subjects. Values are the mean ± SD. *P < 0.05, **P < 0.01.

We next assessed *RUNX2* gene expression in OA and non-OA chondrocytes. *RUNX2* mRNA levels were 34-fold higher in OA chondrocytes than in superficial NOF chondrocytes (p < 0.01), but not compared to deep zone chondrocytes (Fig. 2A). Pyrosequencing analysis of the *RUNX2* promoter revealed that all analyzed CpG sites in the P1 promoter region were significantly demethylated in OA chondrocytes compared to superficial or deep zone NOF chondrocytes (p < 0.01) (Fig. 2B). Furthermore, enhanced *RUNX2* mRNA levels correlated with age (r = 0.534, p = 0.04) (Fig. 2C).

### *RUNX2* gene expression in deep zone NOF chondrocytes is associated with hypomethylation of specific CpG sites in the *RUNX2* promoter

Comparison between superficial and deep zone NOF chondrocytes revealed that the levels of *RUNX2* mRNA were 80-fold higher in the deep zone than in the superficial zone of NOF cartilage (p < 0.01) (Fig. 2A). Pyrosequencing analysis of the *RUNX2* promoter in genomic DNA simultaneously extracted from the same subjects revealed that CpG sites located at +17-bp and −336-bp in the P1 promoter region were significantly hypomethylated in the deep zone compared to the superficial zone chondrocytes (87 ± 3.6% for NOF superficial versus 68 ± 10.7% for NOF deep at + 17-bp CpG; and 91 ± 2.3% for NOF superficial versus 78 ± 6.3% for NOF deep at −336-bp CpG) (p < 0.01) (Fig. 2B).

### *RUNX2* expression is negatively correlated with the percentage methylation of CpG sites in the P1 promoter in OA chondrocytes

**Figure 3:**
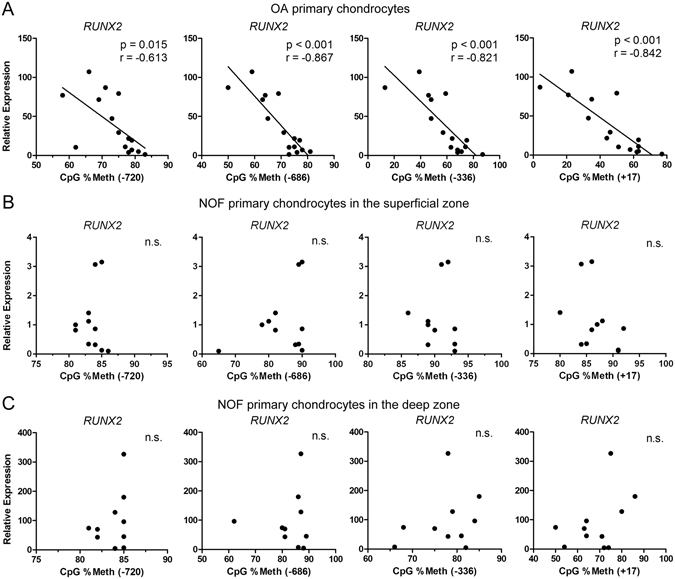
The percentage methylation of CpG sites in the P1 promoter correlates with *RUNX2* gene expression in OA chondrocytes. Spearman’s rank correlation coefficient was used to compare the relative mRNA expression of *RUNX2* and methylation status of the indicated CpG sites in the *RUNX2* P1 promoter in osteoarthritic chondrocytes (**A**) and NOF chondrocytes from the superficial zone (**B**) and deep zone (**C**).

We next assessed whether the levels of *RUNX2* mRNA were correlated with the DNA methylation status of individual CpG sites within the *RUNX2* promoter. As shown in Fig. 3, we found a significant negative correlation between *RUNX2* mRNA levels and the percentage methylation of the CpG sites located at +17-bp, −336-bp, −686-bp and −720-bp in the RUNX2 promoter in OA chondrocytes (Fig. 3A). In contrast, no correlation was observed in superficial zone NOF chondrocytes (Fig. 3B) or in NOF chondrocytes from the deep zone (Fig. 3C).

### Long-term exposure to 5-aza-dC enhances *RUNX2* mRNA levels associated with DNA demethylation at the **−**336-bp CpG site

**Figure 4:**
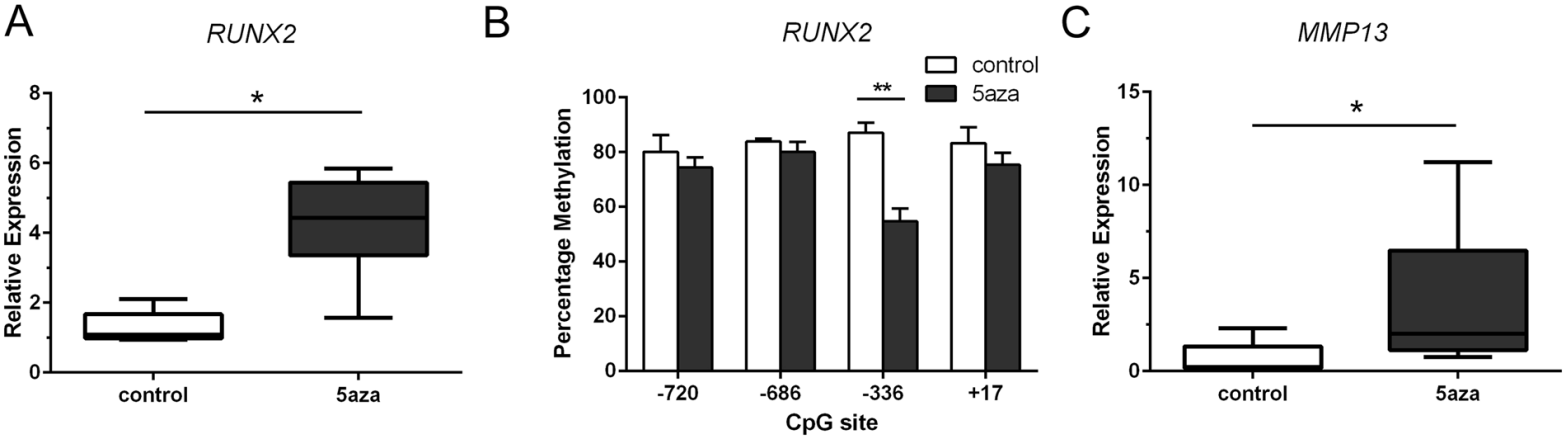
Long-term exposure to 5-aza-dC enhances *RUNX2* gene expression associated with DNA demethylation at the −336-bp CpG site. (**A**) Relative mRNA levels of *RUNX2* were analysed by qRT-PCR and normalized against GAPDH in untreated (control) and 5-aza-dC-treated cultures. (**B**) Percentage methylation of indicated CpG sites in the *RUNX2* P1 promoter was analysed using bisulfite pyrosequencing in the same samples. (**C**) The relative mRNA levels of *MMP13* were analyzed in the same samples. Values represent mean ± SD of 6 independent experiments. *P < 0.05, **P < 0.01.

We further investigated the functional consequences of *RUNX2* promoter demethylation on gene expression using isolated primary chondrocytes *in vitro*. Long-term treatment with 5-aza-dC increased the levels of *RUNX2* mRNA by 3.3-fold compared to untreated cultures (p < 0.01) (Fig. 4A). The CpG site located at −336-bp was significantly demethylated in the same samples (87 ± 3.7% in untreated cultures versus 55 ± 4.6% in 5-aza-treated cultures) (p < 0.01) (Fig. 4B). Similar to previously reported results^48^, long-term treatment with 5-aza-dC enhanced the levels of *MMP13* mRNA by 5.5-fold (p < 0.01) (Fig. 4C).

### RUNX2-driven *MMP13* promoter transactivation is independent of the *MMP13* promoter methylation status

**Figure 5:**
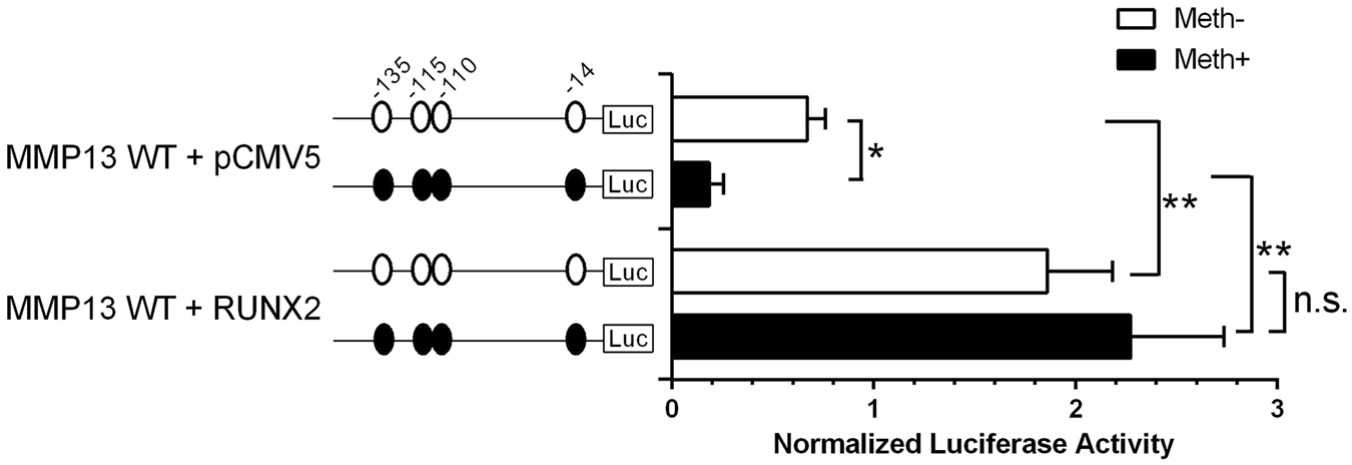
RUNX2-driven *MMP13* promoter transactivation in chondrocytes is not impaired by CpG methylation. *MMP13* promoter activity was analysed by the luciferase reporter assay after transfection of C28/I2 cells with non-methylated or methylated constructs in a CpG-free reporter vector containing the wild-type *MMP13* promoter. Co-transfection with the pCMV-RUNX2 expression vector or the empty control vector (pCMV5) was performed. Values are the mean ± SD of 4 independent experiments. *P < 0.05, **P < 0.01.

To determine whether the transactivation by RUNX2 depends upon the methylation status of the *MMP13* promoter, we co-transfected the expression vector encoding RUNX2 with methylated or unmethylated wild type *MMP13* promoter in CpG-free luciferase reporter constructs. In agreement with our previous reports^28^, DNA methylation significantly decreased the basal activity of the *MMP13* promoter by 3.6-fold. RUNX2 overexpression increased the unmethylated *MMP13* reporter activity by 2.8-fold, but the ability of RUNX2 to transactivate *MMP13* was not affected by the methylation status of the promoter (Fig. 5).

### RUNX2 transcription depends upon the methylation status of specific CpG sites on the proximal promoter

**Figure 6:**
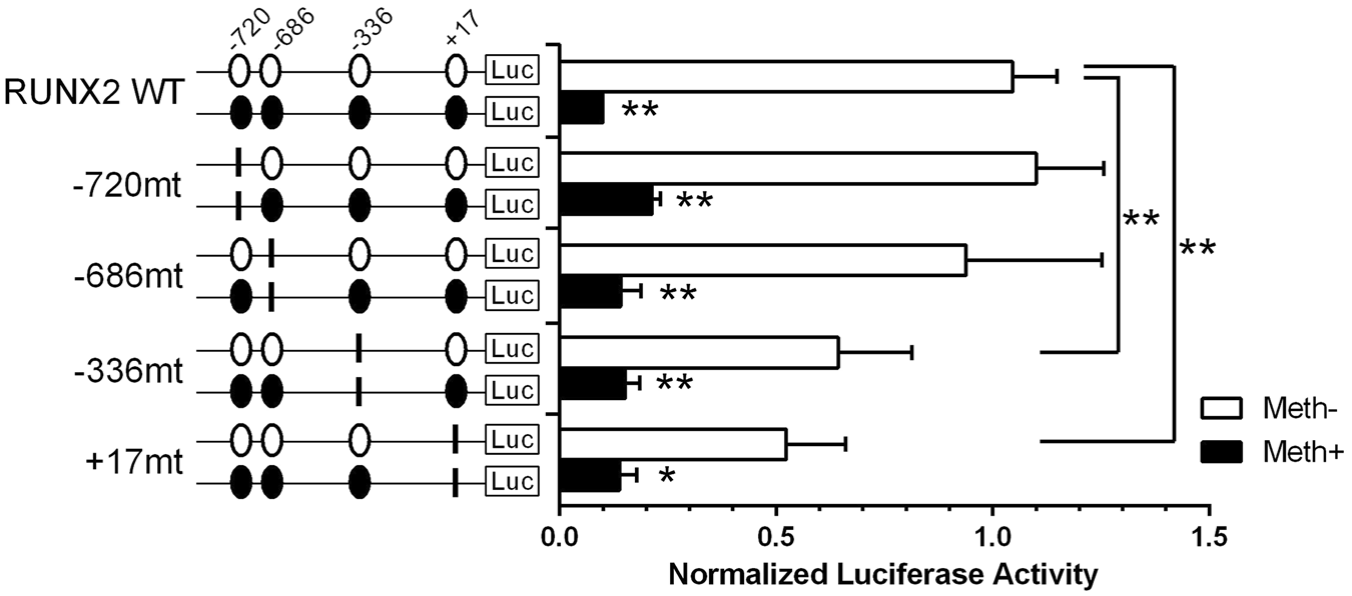
CpG methylation, and specific mutations at the −336-bp and +17-bp CpG sites decrease *RUNX2* promoter activity. Assessment of activities of methylated or non-methylated *RUNX2* promoter constructs containing different mutations was performed by luciferase assay. Point mutations (CG to TG) were created at CpG sites located at −720-bp, −686-bp, −336-bp, and +17-bp. Values are the mean ± SD of 4 independent experiments. *P < 0.05, **P < 0.01.

To further determine the effects of DNA methylation on *RUNX2* promoter activity, and to identify the critical CpG sites involved in promoter regulation, *RUNX2* wild type promoter constructs or promoter constructs containing site-specific CpG mutations were transfected into C28/I2 chondrocytes. CpG methylation decreased the activity of the wild type promoter, as well as the activities of promoter constructs with site-specific CpG mutations. Significantly lower activity was observed in the promoter constructs with mutations at the +17-bp or −336-bp CpG site compared to the non-methylated wild type promoter (Fig. 6).

## Discussion

The current study shows the influence of the methylation status of the transcriptional factor RUNX2 in mediating the *MMP-13* promoter activity, as opposed to the DNA methylation status of the *MMP13* promoter itself. *RUNX2* gene expression significantly correlated with *MMP13* mRNA levels in clinical OA samples. MMP-13 is a major enzyme involved in the pathogenesis of OA that targets types II, IV, and IX collagens, small proteoglycans, perlecan and osteonectin in the articular cartilage^36, 37^. RUNX2 is a key regulator of *MMP13* gene transcription^15, 43, 46, 47^. Therefore, the methylation status of the CpG sites in the *RUNX2* P1 promoter offers a potential target for reducing abnormal *MMP13* gene expression in OA chondrocytes. Importantly, the percentage methylation of CpG sites in the P1 promoter displayed a strong negative correlation with RUNX2 gene expression. In contrast to the *RUNX2* promoter, in this study we did not observe a correlation between the percentage methylation of CpG sites in the *MMP13* promoter with *MMP13* gene expression. Furthermore, *in vitro* methylation of the *MMP13* promoter construct did not inhibit its transactivation by RUNX2. Thus, the methylation status of CpG sites within the *MMP13* proximal promoter did not influence RUNX2-driven *MMP13* transactivation, in contrast to HIF-2alpha-driven *MMP13* promoter activity, as we have reported previously^28^. Crucially, CpG methylation is likely to be a contributory factor through modulation of binding of trans-acting factors, but also through acting together with histone-modifying, chromatin-remodeling activities and other transcriptional regulatory elements.

It is known that chondrocytes in the superficial and deep zones display different gene expression patterns^49^. Da Silva *et al*. reported that superficial chondrocytes in aged NOF cartilage express comparable proteases and display characteristics typical of the deep zone cartilage of young healthy cartilage^50^. In the current study, enhanced *MMP13* gene expression together with hypomethylation of the CpG site at −14-bp CpG was observed in the superficial chondrocytes, in agreement with Da Silva. Furthermore, our results showing enhanced *RUNX2* mRNA levels in deep zone chondrocytes are consistent with the findings of Wang *et al*., who reported, based on immunocytochemical studies, that RUNX2 was rarely observed in surface chondrocytes, but was detected frequently in deep zone chondrocytes^47^. As for the epigenetic status, Erura *et al*.^51^ reported that CpG islands in the *RUNX2* promoter are hypomethylated in the superficial and deep zone chondrocytes. In contrast, the current studies indicate that two discrete CpG sites (−336-bp and +17-bp) in the P1 promoter are hypomethylated to a significantly higher extent in the chondrocytes of the deep zone compared to the superficial chondrocytes. Additionally, mutations (CG to TG) created at −336-bp and +17-bp CpG sites significantly decreased *RUNX2* promoter activity. Furthermore, 5-aza-dC treatment reduced methylation at the −336-bp CpG site in the *RUNX2* P1 promoter associated with higher *RUNX2* promoter activity. Thus, the methylation status of specific CpG sites (−336-bp and +17-bp) rather than CpG islands in the promoter region are pivotal in the epigenetic regulation of *RUNX2*. In a study from Den Hollander *et al*., the preserved cartilage area and the OA-affected area in the same joint were compared and transcriptionally active CpG sites in *RUNX2* were not detected^52^. In contrast, other genome-wide DNA methylation analysis studies, adopting a similar approach, clearly demonstrated markedly decreased methylation of the RUNX2 gene in OA chondrocytes^53, 54^, which is consistent with our study.

Previous studies have demonstrated that the DNA methylation status of the regulatory sequences of several key genes in OA chondrocytes largely differs compared to healthy chondrocytes^53–55^. Epigenetic changes in OA are characterised by CpG hypomethylation in the promoter regions of catabolic genes^28, 38, 56^ and hypermethylation of at least one anabolic gene^27^. In addition, we have shown previously that the loss of methylation in CpG sites in a specific NF-κB-responsive enhancer element is responsible for enhanced *NOS2* promoter activity in OA^29^, indicating that the methylation status of the promoter or enhancer of a particular gene is related to the expression level of that gene. Thus the methylation status of the *RUNX2* promoter ultimately determines the amount of RUNX2 protein available for driving *MMP13* promoter activity and gene transcription. Of interest is the association of RUNX2 with the regulation of other genes such as *COL10A1* and other hypertrophy markers in the deep zone that may drive cartilage calcification^4, 6, 8–13^.

In conclusion, *RUNX2* promoter activity is enhanced by de-methylation of specific CpG sites in the P1 promoter. The increased availability of RUNX2 for binding to and activating the *MMP13* promoter is likely responsible in part for the increased gene expression of this cartilage-degrading proteinase in human OA chondrocytes, at least in the deep zone. These findings offer a unique target for pharmacological interventions that modulate methylation status of genes associated with cartilage pathology in OA and, potentially, other arthritic diseases.

## Methods

### Chondrocyte isolation

Human articular cartilage was dissected from femoral heads obtained from patients undergoing hemiarthroplasty for fractured neck of femur (NOF, controls, 7 men and 4 women with a mean ± SD age of 80.5 ± 7.7) or OA patients undergoing total hip arthroplasty (OA, 7 men and 8 women with a mean ± SD age of 66.7 ± 12.5). The cartilage from #NOF patients is widely used as a suitable non-OA control^50^. Samples were obtained with full patient consent and prior approval of the local Institutional Review Board. The OARSI score^57^ ranged from 3 to 4 in all OA patients examined. Cartilage was dissected within 6 hours of surgery and chondrocytes, were isolated as detailed previously^50, 56^. In brief, samples were obtained from the superficial and deep zones of cartilage from patients with NOF for isolation of non-OA/healthy chondrocytes, whereas full thickness OA chondrocytes were taken adjacent to weight-bearing areas of OA femoral heads (lacking surface zones). The superficial half of the #NOF cartilage was harvested as the superficial zone and remaining half as the deep zone. Cartilage samples were cut into small fragments and digested with 10% trypsin (Lonza) in PBS for 30 min, followed by sequential digestions in 1 mg/ml of hyaluronidase (Sigma-Aldrich) in PBS for 15 min, and in 10 mg/ml of collagenase B (Roche Applied Science) in DMEM/F12 medium (Life Technologies) for 12–15 hours at 37 °C. Isolated chondrocytes from 11 NOF samples and 15 OA samples were directly used for extraction of genomic DNA and total RNA. Chondrocytes isolated from 6 NOF patients were placed in culture and used for *in vitro* experiments.

### Chondrocyte culture

Following isolation, non-OA chondrocytes were divided into two groups: i) untreated controls and ii) 5-azadeoxycytidine (5-aza-dC) treatment. Chondrocytes were cultured at a density of 2 to 4 × 10^5^ cells/25-cm^2^ flask in 5 ml of DMEM/F12 supplemented with 5% fetal calf serum, 1% insulin-transferrin-selenium, 100 units/ml of penicillin and 100 μg/ml of streptomycin, and 100 μg/ml of ascorbic acid, in a controlled atmosphere of 5% CO_2_ at 37 °C. For 5-aza culture, the cells were cultured with 2 μM 5-aza-dC, a cytidine analog that inhibits the activity of DNMT-1, which induce non-specific loss of DNA methylation during cell division. The histone deacetylase inhibitor trichostatin A (300 nM) was added at the first treatment to facilitate access of 5-aza-dC^58^. The primary cultures were maintained for 5 weeks until cells reached confluence, as described previously^27^.

### DNA and RNA extraction and quantitative reverse transcription–polymerase chain reaction (qRT-PCR)

Total RNA and genomic DNA were extracted simultaneously from the isolated chondrocytes using AllPrep DNA/RNA Mini kit (Qiagen). RNA was immediately reverse transcribed with SuperScript VILO cDNA Synthesis Kit (Life Technologies). Relative quantification of gene expression was performed with an ABI Prism 7500 detection system (Applied Biosystems). A 20-μl reaction mixture was prepared in triplicate, containing 1 μl of complementary DNA, 10 μl of Power SYBR Green PCR Master Mix (Applied Biosystems), and 250 nM of each primer. Thermal cycler conditions included an initial activation step at 95 °C for 10 minutes, followed by a 2-step PCR program of 95 °C for 15 seconds and 60 °C for 60 seconds for 40 cycles. The 2^−ΔΔCt^ method was used for relative quantification of mRNA, and the data were normalized to GAPDH mRNA. The primers used for qRT-PCR were designed using Primer Express software (version 3.0; Applied Biosystems), and the sequences are shown in Table 1.

**Table 1:** (a) Primer sequences for qRT-PCR, (b) Primer sequences for pyrosequencing, and (c) site directed mutagenesis.

Amplicon ID (length, bp) [target CpG]	Sequence (5′ to 3′)
**(a)**	
GAPDH (108)	F (CCAGGTGGTCTCCTCTGACTTC)
	R (TCATACCAGGAAATGAGCTTGACA)
RUNX2 (78)	F (GTAGATGGACCTCGGGAACC)
	R (GAGGCGGTCAGAGAACAAAC)
MMP13 (71)	F (TTAAGGAGCATGGCGACTTCTAC)
	R (CCCAGGAGGAAAAGCATGAG)
Osterix (OSX) (75)	F (ATGGGCTCCTTTCACCTG)
	R (GGGAAAAGGGAGGGTAATC)
**(b)**	
RUNX2-Pyro-0 (107)	F (GTTTTTGTTTTTTTGGATTGTGTGA)
	R (CCAAAAACTTCTTACTATCCTCCTAA)
[+17]	S (TGGATTGTGTGAATGT)
RUNX2-Pyro-1 (60)	F (AGAGGAGGTAAAAAGGTAGAGG)
	R (TCTACAATTAAAAACTTTCCTTTCTACTCCC)
[−336]	S (GGTAAAAAGGTAGAGGTTG)
RUNX2-Pyro-2 (175)	F (TGGTTGTTATGAAAGTGTTAGTT)
	R (CCCTATCATTCATTTTTTTAAAATCTTC)
[−686, −720]	S (TTTGGGTATTTTTTTATAAATTTT)
MMP13-Pyro-1 (216)	F (AATTAGTATTAAGTTTTTTTTTATGGAAGT)
	R (TTCAACAAAATCTCAAAACCCATCTAA)
[−323, −343]	S1 (AAATTTTTTTTTTTTTACCTTCTAT)
[−225]	S2 (CTCAAAACCCATCTAAC)
MMP13-Pyro-5 (145)	F (GGTTTTGAGATTTTGTTGAAATAAGAGA)
	R (ATAAATAAATTTCCACTTCCCAATCAC)
[−135]	S1 (ATATTTTTTTTAAATTTTATTATAAATTA)
[−115, −110]	S2 (GGAGGGAAAAGAAAAAGT)
MMP13-Pyro-6 (151)	F (GTATGTTTATTTTTAAGTGATTGGGAAGTG)
	R (AACAACCAAAACCCCTAAATACA)
[−14]	S (AGGTTTATAAAAGTAAAGGTAATT)
**(c)**	
RUNX2-Mut1 [−720*]^#^	F (CTAGTTTATTATCAATCTATTAGATGGCaGCCTTTACAATAAAGATTAAATGTAATG)
	R (CATTACATTTAATCTTTATTGTAAAGGCtGCCATCTAATAGATTGATAATAAACTAG)
RUNX2-Mut2 [−686*]^#^	F (GGGCATTCTTTTACAAATTTTAAATCACaTCTGTCTAGTTTATTATCAATCTATTAG)
	R (CTAATAGATTGATAATAAACTAGACAGAtGTGATTTAAAATTTGTAAAAGAATGCCC)
RUNX2-Mut3 [−336*]^#^	F (TTTCCTTTCTACTCCCCaCTCAACCTCTGCCTTTT)
	R (AAAAGGCAGAGGTTGAGtGGGGAGTAGAAAGGAAA)
RUNX2-Mut4 [+17*]^#^	F (GGTTGTTTGTGAGGCaAATGAAGCATTCACACAATCCAAAAAAGC)
	R (GCTTTTTTGGATTGTGTGAATGCTTCATTtGCCTCACAAACAACC)

*Location of mutated CpG, ^#^Lowercase letters indicate a mutated base, F: forward; R: reverse; S: sequencing.

### Bisulfite pyrosequencing

Genomic DNA extracted from each sample was treated with sodium bisulfite to convert unmethylated cytosine in CpG sites to uracil using the EZ DNA Methylation-Gold Kit (Zymo Research Corporation), as described^29^. After bisulfite treatment, PCR was performed with Premium PCR Supermix High Fidelity (Invitrogen). The percentages of DNA methylation in the *RUNX2* and *MMP13* promoters were quantified using PyroMark MD (Qiagen), as described^27–29^. The primers used are detailed in Table 1. All primers were designed using Pyrosequencing Assay Design Software (Qiagen).

### Plasmid constructions

The *RUNX2* P1 promoter construct (spanning −788/+32 and containing four CpG sites) and the *MMP13* promoter construct (spanning −214/+14 and containing four CpG sites) were generated by PCR amplification, as described previously^28^, using genomic DNA from human articular chondrocytes as template and the following PCR primers for the *RUNX2* promoter: 5′-ATGGGATCCAGATCTTCAAACTAGGCATGAGA-3′ (forward) and 5′-ATACCATGGGGTTGTTTGTGAGGCGAA-3′ (reverse), and for the *MMP13* promoter: 5′-CCGACTAGTATTTTGCCAGATGGGTTTTG-3′ (forward) and 5′-CCGAAGCTTCCTGGGGACTGTTGTCTTT-3′ (reverse). Underscore indicates BamHI, NcoI, SpeI, and HindIII restriction sites, respectively. The resultant PCR products were digested with the restriction enzymes and transferred into the multiple cloning site of a pCpGfree-Luc vector using Rapid DNA Ligation Kit (Thermo Scientific). The vector lacks CpG sites within the whole vector sequence and was generated according to the literature^59^, as described^28^. Point mutations at CpG sites in the *RUNX2* promoter constructs were generated by converting CG to TG using the QuickChange II Site-Directed Mutagenesis Kit (Agilent Technologies). The primers used for mutagenesis were designed using QuickChange Primer Design (Agilent Technologies) and described in Table 1. RUNX2 promoter constructs with mutation (CG to TG) of CpG sites located at +17-bp, −336-bp, −686-bp and −720-bp from the TSS were generated according to the manufacturer’s instructions. Construct sequences were confirmed by DNA sequencing using the SmartSeq system (Eurofins Genomics).

### *In vitro* methylation, transfection and luciferase assay

Reporter constructs were methylated using CpG Methyltransferase M.Sssl (New England Biolabs). Complete methylation was verified by plasmid DNA bisulfite modification and pyrosequencing using specific primers. The immortalized human chondrocytes, C28/I2, were seeded at a density of 30,000 cells per well in 24-well plates, cultured in complete DMEM/F12 + 5% FBS + ITS + A2P + P/S overnight, and transfected with a mixture of 500 ng of luciferase reporter vector and 1 ng of pRL-TK Vector (Promega), using FuGENE HD *in vitro* Transfection Reagent (Promega) in serum-free conditions. Transfected C28/I2 cells were cultured for an additional period of 48 hours prior to harvest. Cell lysates were assayed for Firefly and Renilla luciferase activities using the Dual-Luciferase Reporter Assay System on a Varioskan Flash Multimode Reader (Thermo Scientific). Firefly luciferase activity of each transfection was normalized against Renilla luciferase activity. The RUNX2 expression vector was used in co-transfection experiments, and the empty pCMV5 backbone served as a control. Reactions were performed in duplicate, and each experiment was repeated 4 times.

### Statistical analysis

Statistical analysis was performed using SPSS Statistics (version 21.0; IBM). Wilcoxon rank-sum test was used to compare mRNA levels and CpG percentage methylation. Bonferroni correction was used for multiple comparisons. Spearman’s rank correlation coefficient was used to analyse correlative relationships. The Kruskal-Wallis test and Newman-Kuels multiple comparisons test were used to analyse the luciferase reporter assays. Values of P ≤ 0.05 were considered significant.
